# The Gut Hormones PYY_3-36_ and GLP-1_7-36 amide_ Reduce Food Intake and Modulate Brain Activity in Appetite Centers in Humans

**DOI:** 10.1016/j.cmet.2011.09.010

**Published:** 2011-11-02

**Authors:** Akila De Silva, Victoria Salem, Christopher J. Long, Aidan Makwana, Rexford D. Newbould, Eugenii A. Rabiner, Mohammad A. Ghatei, Stephen R. Bloom, Paul M. Matthews, John D. Beaver, Waljit S. Dhillo

**Affiliations:** 1 Section of Investigative Medicine, Division of Diabetes, Endocrinology and Metabolism, Imperial College London, London W12 0NN, UK; 2 Department of Experimental Medicine, Imperial College London, London W12 0NN, UK; 3 Centre for Neuroscience, Department of Medicine, Imperial College London, London W12 0NN, UK; 4 GlaxoSmithKline Clinical Imaging Centre, Hammersmith Hospital, London W12 0NN, UK

## Abstract

Obesity is a major public health issue worldwide. Understanding how the brain controls appetite offers promising inroads toward new therapies for obesity. Peptide YY (PYY) and glucagon-like peptide 1 (GLP-1) are coreleased postprandially and reduce appetite and inhibit food intake when administered to humans. However, the effects of GLP-1 and the ways in which PYY and GLP-1 act together to modulate brain activity in humans are unknown. Here, we have used functional MRI to determine these effects in healthy, normal-weight human subjects and compared them to those seen physiologically following a meal. We provide a demonstration that the combined administration of PYY_3-36_ and GLP-1_7-36 amide_ to fasted human subjects leads to similar reductions in subsequent energy intake and brain activity, as observed physiologically following feeding.

## Highlights

► Coadministration of PYY_3-36_ and GLP-1_7-36 amide_ reduces food intake and brain activity ► The reduction in brain activity is notable in areas that control appetitive behavior ► Changes in food intake and brain activity are similar to that observed after a meal

## Introduction

Obesity poses a major public health issue in modern societies. Pharmacological treatments have been disappointing and have been limited by CNS and cardiovascular side effects. Promisingly, anorectic gut hormones, including peptide YY (PYY) and glucagon-like peptide 1 (GLP-1), have recently emerged as potential therapeutic targets for obesity.

Both PYY and GLP-1 are released postprandially into the circulation by the enteroendocrine L cells of the gut. Peripheral administration of PYY_3-36_ to humans leads to marked inhibition of food intake (Batterham et al., 2002; Sloth et al., 2007). A range of studies has also demonstrated that peripheral administration of GLP-1_7-36 amide_ to humans leads to a dose-dependent reduction in appetite and ad libitum energy intake (Verdich et al., 2001). Recently there has been interest in investigating how these satiety factors act together to physiologically regulate postprandial satiety (Neary et al., 2005). It is likely that these hormones act via the CNS (Batterham et al., 2002; Turton et al., 1996), although the mechanisms by which GLP-1, in addition to PYY and GLP-1 in combination, mediate their anorectic effect in humans are unknown.

Blood oxygen level-dependent (BOLD) functional magnetic resonance imaging (fMRI) has recently been used as a tool to investigate the changes in brain activity associated with differences in nutritional status in humans. Activity of reward systems in the brain is increased in the fasted state compared to the fed state with presentation of food-relevant stimuli (LaBar et al., 2001). However, there are only a few reports on the use of fMRI for characterization of brain activity following the systemic administration of hormones affecting appetite in humans (Baicy et al., 2007; Batterham et al., 2007; Farooqi et al., 2007; Malik et al., 2008; Rosenbaum et al., 2008). Intravenous infusion of PYY_3-36_ to human subjects modulates activity in brain regions mediating appetitive behavior and leads to reduced food intake (Batterham et al., 2007). However, there have been no human fMRI studies investigating effects of administration of GLP-1 or coadministration of PYY and GLP-1 on brain activity in humans.

In this work, we have used BOLD fMRI to investigate the changes in brain activity following PYY_3-36_ or GLP-1_7-36 amide_ (either as single or combined administration) in fasted healthy human subjects and compared the effects to those seen naturally following a meal. We demonstrate that following a meal, there is a reduction in subsequent energy intake and BOLD fMRI signal in several brain regions that have been previously implicated in responses to food reward. We show that coadministration of PYY_3-36_ and GLP-1_7-36 amide_ to fasted human subjects results in a reduction in BOLD fMRI signal and in subsequent energy intake similar to that occurring following feeding. Together, these findings provide evidence in humans that PYY_3-36_ and GLP-1_7-36 amide_ in combination result in changes in brain activity, which are similar to those observed after a meal. In combination, PYY_3-36_ and GLP-1_7-36 amide_ may well act via a final common pathway as key mediators of postprandial satiety.

## Results

### Both Feeding and the Infusion of PYY_3-36_ and GLP-1_7-36 amide_ Reduce Subsequent Energy Intake

**Figure 1:**
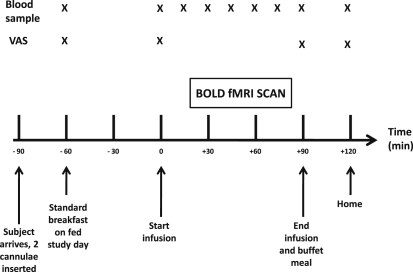
Study Protocol Following an overnight fast, 16 healthy, normal-weight subjects each received the following interventions, in random order, over 5 separate study days in a single-blinded fashion: (1) 90 min saline infusion (fasted saline, control visit); (2) standard breakfast, then 90 min saline infusion (fed saline); (3) 90 min PYY_3-36_ infusion at 0.3 pmol/kg/min; (4) 90 min GLP-1_7-36 amide_ infusion at 0.8 pmol/kg/min; (5) 90 min combined PYY_3-36_ and GLP-1_7-36 amide_ infusion, at 0.3 pmol/kg/min and 0.8 pmol/kg/min, respectively. On each visit, subjects underwent a 60 min fMRI scan, which commenced 20 min after the start of the infusion. During the fMRI scan, a picture processing task was performed where images of food and nonfood were shown. The mean percent change in BOLD signal in prespecified brain ROIs when viewing images of food compared to nonfood were determined for each study day. An ad libitum buffet meal was served immediately after the infusion on all study days in order to measure energy intake. Blood sampling (for PYY_3-36_ and GLP-1_7-36 amide_) and assessments of appetite (based on VAS after direct questioning) was performed. One subject was excluded from analysis due to excessive head movement during the scans, leaving data from 15 subjects for analysis.

**Figure 2:**
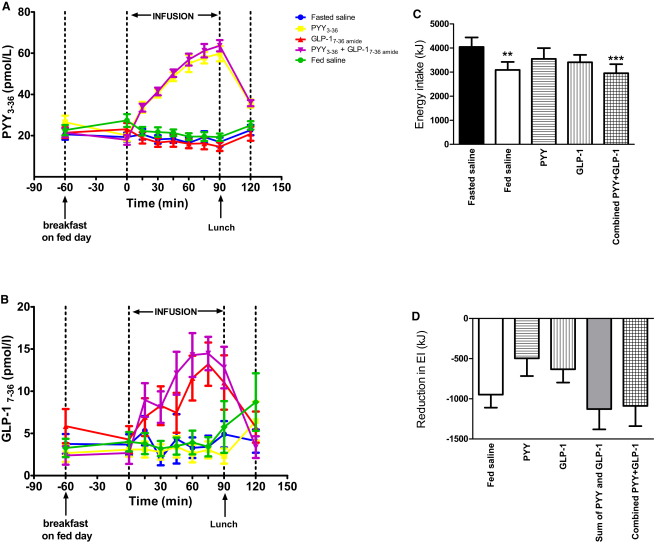
Analysis of Plasma PYY_3-36_ and GLP-1_7-36 amide_ Levels and Mean Ad Libitum Energy Intake (A–D) On each study visit, the hormone infusion was administered between t = 0 and t = 90 min. Shown are plasma PYY_3-36_ levels (A) and plasma GLP-1_7-36 amide_ levels (B). Data are shown as mean ± SEM for 15 subjects. An ad libitum buffet meal was served immediately after the infusion in order to measure energy intake on all study days. Shown are the energy intake during the buffet meal following each infusion (C) and the reduction in energy intake during the buffet meal for each infusion versus the fasted saline infusion and also the sum of the individual effects of PYY_3-36_ and GLP-1_7-36 amide_ in reducing energy intake (sum of PYY and GLP-1) (D). Data are shown as mean ± SEM, grouped for 15 subjects. ^∗∗^p < 0.01 versus fasted saline. ^∗∗∗^p = 0.0001 versus fasted saline. Abbreviations: EI, energy intake; kJ, kilojoules; PYY, PYY_3-36_; GLP-1, GLP-1_7-36 amide_. See also Figure S1.

Following saline, PYY_3-36_, or GLP-1_7-36 amide_ (either as single or combined administration) in fasted human subjects, we measured ad libitum energy intake during a buffet lunch (Figure 1). Plasma levels of PYY_3-36_ or GLP-1_7-36 amide_, which are the active forms of these hormones, increased on infusion (Figures 2A and 2B). We compared the effects of PYY_3-36_ and GLP-1_7-36 amide_ to those seen naturally following a breakfast meal. Consumption of a standard breakfast by subjects before saline infusion led to a 23.5% ± 8.3% reduction in energy intake during the subsequent ad libitum buffet lunch compared to when subjects received only saline infusion. Infusion of PYY_3-36_, GLP-1_7-36 amide_, or combined PYY_3-36_ and GLP-1_7-36 amide_ to fasted subjects resulted in 12.3% ± 11.2%, 15.7% ± 7.5%, and 27.0% ± 9.4% reductions in energy intake, respectively, compared to when subjects received saline infusion (Figure 2C). There were no order effects on energy intake across visits (data not shown).

We compared the additive effects of single infusions of PYY_3-36_ and GLP-1_7-36 amide_ with combined infusion of PYY_3-36_ and GLP-1_7-36 amide_ on energy intake at the ad libitum lunch (Figure 2D). The summed reduction in energy intake by each of the single hormone infusions was comparable with the reduction after the combined infusion.

### Effects of Feeding or Infusion of PYY and GLP-1 on Appetite

Visual analog scores (VAS) of appetite by subjects confirmed that consumption of the standard breakfast or combined infusion of PYY_3-36_ and GLP-1_7-36 amide_ reduced hunger and increased their sense of “fullness” (Figure S1). There was no correlation between nausea and energy intake during the ad libitum buffet meal (p = 0.23, r^2^ = 0.02, Figure S1F).

### Brain Activation with Presentation of Food-Salient Visual Stimuli

**Figure 3:**
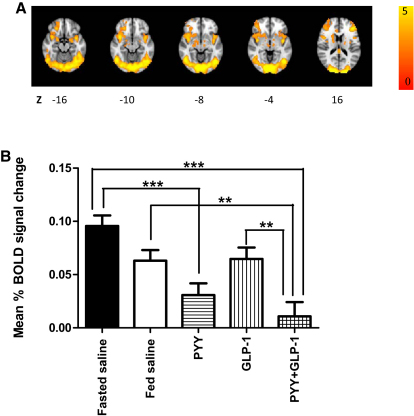
Modulation of BOLD Signal across Brain ROIs (A and B) During each infusion, a BOLD fMRI scan was performed, incorporating a picture processing task where images of food and nonfood were shown. For the fasted saline and fed saline infusions, a whole-brain map of brain regions activated (z-statistics) by food images (compared with nonfood images) is shown (A). Z indicates distance (mm) superior or inferior to the intercommissural plane in standard stereotactic space. Clusters were thresholded by means of parametric testing at the level of spatially contiguous suprathresholded clusters, simultaneously controlling the family-wise probability of type 1 error at p < 0.05, corrected. The mean percent BOLD signal change (food images minus nonfood images) across all six ROIs studied (amygdala, insula, caudate, nucleus accumbens, OFC, and putamen), is shown for each of the interventions (B). Data are shown as mean ± SEM, grouped for 15 subjects. Abbreviations: PYY, PYY_3-36_; GLP-1, GLP-1_7-36 amide_; ^∗∗^p < 0.01; ^∗∗∗^p < 0.001.

We assessed brain activation with presentation of food-salient images using BOLD fMRI. We a priori chose to test six regions of interest (ROIs) (amygdala, caudate, insula, nucleus accumbens, orbitofrontal cortex [OFC], and putamen) based on previous research in the field (Batterham et al., 2007; Farooqi et al., 2007; Fletcher et al., 2010; LaBar et al., 2001; Malik et al., 2008). We found that when subjects infused with saline (in either the fasted or fed state) viewed images of food, there was greater BOLD signal in these regions compared with when nonfood images were viewed (Figure 3A). A whole-brain cluster-wise analysis supported the selection of these ROIs (Table S1).

### Feeding, PYY, and GLP-1 Administration Reduce Brain Activation by Food Images

We first tested the effects of feeding, PYY_3-36_, GLP-1_7-36 amide_, and combined PYY_3-36_ and GLP-1_7-36 amide_ on changes in mean percent BOLD signal in the total brain volume represented by the six a priori selected ROIs (Figure 3B). When fasted subjects viewed images of food compared with when they viewed images of nonfood, there was a significant reduction (p < 0.001) in the mean percent BOLD fMRI signal change after administration of PYY_3-36_ or after coadministration of PYY_3-36_ and GLP-1_7-36 amide_ compared to when subjects were fasted and received saline infusion (control). There was a nonsignificant reduction in the mean percent BOLD signal change in the selected ROIs following feeding or after GLP-1_7-36 amide_ administration compared to when subjects were fasted and received saline infusion.

There was no observed correlation between changes in nausea ratings and the mean percent change in BOLD signal for any of the interventions (data not shown). Similarly, there was no observed correlation between the mean percent change in BOLD signal and subsequent energy intake during the ad libitum buffet lunch for any of the interventions (data not shown).

**Figure 4:**
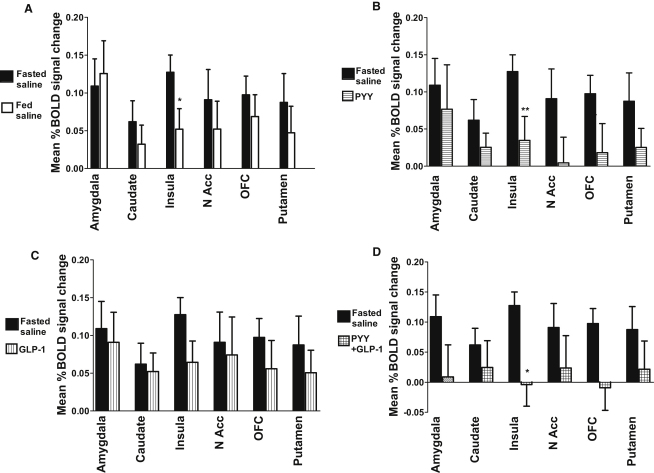
Modulation of BOLD Signal across Brain ROIs by Feeding or Either Individual or Combined Gut Hormone Infusions (A–D) Subjects underwent a 90 min infusion of saline (fasted saline) as a control. They also had four further infusions: saline after a standard breakfast (A), PYY_3-36_ after an overnight fast (B), GLP-1_7-36 amide_ after an overnight fast (C), and combined PYY_3-36_ + GLP-1_7-36 amide_ after an overnight fast (D). During each infusion, a BOLD fMRI scan was performed, incorporating a picture processing task where images of food and nonfood were shown. The mean percent BOLD signal change when subjects viewed images of food compared with when they viewed images of nonfood is shown for each of the infusions administered as a comparison with the fasted saline infusion: ^∗^p = 0.015 for fed saline < fasted saline and p = 0.012 for PYY + GLP-1 < fasted saline. ^∗∗^p = 0.005 for PYY < fasted saline. Data are shown for individual ROIs (amygdala, insula, caudate, nucleus accumbens [N Acc], OFC, and putamen), combined for left and right hemispheres and grouped for 15 subjects, shown as mean ± SEM. Abbreviations: PYY, PYY_3-36_; GLP-1, GLP-1_7-36 amide_. See also Figure S2.

We next examined the mean percent change in BOLD signal (when subjects viewed images of food compared to when they viewed images of nonfood) after feeding, PYY_3-36_, GLP-1_7-36 amide_, or combined PYY_3-36_ and GLP-1_7-36 amide_ infusion across the individual a priori selected ROIs (left and right hemisphere data combined). We compared this to the mean percent change in BOLD signal when subjects were fasted and received saline (control). There were similar reductions in mean percent change in BOLD signal for all of the ROIs studied following feeding or gut hormone administration, compared to when subjects were fasted and received saline (Figure 4). In addition, individual analysis of left and right hemisphere ROIs showed that reductions in mean percent BOLD signal change for PYY_3-36_ versus fasted saline in the left nucleus accumbens (p = 0.01) and the left OFC (p = 0.02) and for GLP-1_7-36 amide_ versus fasted saline in the right insula (p = 0.049) were significant.

The reduction in mean percent BOLD signal change with the combined infusion of PYY_3-36_ and GLP-1_7-36 amide_ was similar to the summed reduction in mean percent BOLD signal changes after individual administrations of the two hormones (Figure S2).

## Discussion

We found that coadministration of PYY_3-36_ and GLP-1_7-36 amide_ to subjects fasted overnight resulted in similar reductions in ad libitum energy intake during a subsequent buffet lunch as in subjects who had been fed a standard breakfast. Single infusions of either PYY_3-36_ or GLP-1_7-36 amide_ resulted in smaller reductions in ad libitum energy intake. The summed effects of the single hormones (PYY_3-36_ or GLP-1_7-36 amide_) were similar to the reduction in energy intake following combined PYY_3-36_ and GLP-1_7-36 amide_ infusion; coadministration of PYY_3-36_ and GLP-1_7-36 amide_ did not appear to lead to loss of effect of either individual hormone. We have shown that combined gut hormone infusion of PYY_3-36_ and GLP-1_7-36 amide_ results in levels of modulation of brain activity similar to those observed following their summed effects after single infusion. This is consistent with expectations based on endogenous physiological responses observed postprandially, whereby several anorectic gut hormones are cosecreted and are thought to act together to limit further food intake.

We have presented data showing the modulation of BOLD fMRI signal by feeding within six a priori selected ROIs (amygdala, caudate, insula, nucleus accumbens, OFC, and putamen). These were based on previous studies (Batterham et al., 2007; Farooqi et al., 2007; Fletcher et al., 2010; LaBar et al., 2001; Malik et al., 2008) and on our derived functional group activation maps to inform the particular choice of anatomical masks of the task-related regions. As predicted, our data demonstrated that in each of the six brain reward ROIs, viewing images of food was associated with a greater BOLD fMRI signal than viewing nonfood images in the fasted state. Consumption of a standard breakfast reduced this brain activation, extending earlier observations (LaBar et al., 2001). Activation of the insula was most sensitive to modulation by consumption of a meal.

We then assessed the change in BOLD signal following infusion of PYY_3-36_ and GLP-1_7-36 amide_, either singly or in combination, in fasted subjects. When PYY_3-36_ alone was infused in the fasted state, the pattern of signal change in brain reward ROIs was similar to that observed after feeding, with consistent reductions in mean percent BOLD signal change across all brain reward ROIs. Reductions in signal change were prominent in the insula, left nucleus accumbens, and left OFC. Changes in OFC activation were also reported in an earlier investigation of PYY effects (Batterham et al., 2007). In that study, subsequent food intake was predicted by the modulation of OFC signal with PYY_3-36_ infusion, but not with saline infusion after fasting, when subsequent food intake was better correlated with changes in hypothalamic signal. It was postulated that the presence of PYY_3-36_ switches regulation of food intake from a homeostatic brain region (hypothalamus) to a hedonic region (OFC). However, differences in methodology used (our study used task-activated BOLD fMRI data, whereas Batterham et al. tested for changes in resting-state BOLD fMRI) confounds direct comparisons between the studies.

Analysis of the effects of GLP-1_7-36 amide_ on human brain activation revealed that infusion of GLP-1_7-36 amide_ alone led to consistent reductions in mean percent BOLD signal change compared with fasted saline across all of the selected ROIs, with the greatest change in the right insula.

We also characterized the combined effects of infusion of PYY_3-36_ and GLP-1_7-36 amide_ on brain activity. We found consistent reductions in mean percent BOLD signal change relative to infusion of saline across all of the selected ROIs, similar to those observed following a meal. As with GLP-1_7-36 amide_ alone, the insula appeared particularly sensitive to hormonal modulation. Consistent with effects on ad libitum energy intake, summation of the effect of each single hormone in reducing mean percent BOLD signal change was comparable with the reduction in mean percent BOLD signal change after the combined infusion of PYY_3-36_ and GLP-1_7-36 amide_. This provides evidence in humans that the actions of PYY_3-36_ and GLP-1_7-36 amide_ on brain responses to food-salient stimuli are additive, explaining the way in which gut hormones cosecreted physiologically after meals may work in concert to limit further food intake and cause satiety. Furthermore, the lack of any obvious differential activation pattern between PYY_3-36_ and GLP-1_7-36 amide_ suggests that these hormones may be acting at the level of higher reward centers via a final common pathway.

We used the full dimension of the functional imaging dataset to generate a priori knowledge of where activations were expected in the picture processing task—an approach that, as with all group-based fMRI analyses, possesses the inherent limitation of assuming that (during ROI analysis) the estimated activated areas are the same for each contributing individual subject. Furthermore, because the whole brain map of brain regions activated by food images (compared with nonfood images) was generated from the fasted saline and fed saline visits, a further limitation lies in the assumption that these same brain regions would be modulated by infusion of PYY_3-36_ and GLP-1_7-36 amide_.

It is of interest to consider our findings in relation to other fMRI studies that have explored the neuroendocrinology of appetite. In two congenitally leptin-deficient human subjects, daily subcutaneous leptin replacement reduced BOLD fMRI activation when viewing food versus nonfood images in the nucleus accumbens, caudate, putamen, and globus pallidus (Farooqi et al., 2007). We found similar activation changes following infusion of PYY_3-36_ and GLP-1_7-36 amide_. However, in our study the most significant reductions in mean percent BOLD signal change following infusion of PYY_3-36_ and GLP-1_7-36 amide_ were in the insula, left nucleus accumbens, and left OFC. This implies that leptin, compared with PYY and GLP-1, modulates distinct neural networks. We speculate that this may be related to the longer-term anorectic signaling by the adipokine leptin, relative to the more acute anorectic effects of postprandially released gut hormones.

Our results are also interesting in relation to a previous study investigating the effects of ghrelin, the only known gut hormone which acutely increases food intake, on brain activity in normal-weight humans (Malik et al., 2008). Intravenous ghrelin infusion increased BOLD activation when subjects viewed images of food compared to when they viewed images of nonfood in the amygdala, OFC, insula, visual areas, and striatum. Intravenous ghrelin infusion also resulted in increased food intake compared with saline infusion. By contrast, in our study, the gut hormones PYY_3-36_ and GLP-1_7-36 amide_, which acutely inhibit food intake, resulted in a reduction in mean percent BOLD signal change in these ROIs. Collectively, our results and those of Malik et al. suggest that certain brain regions form CNS networks, which when activated by ghrelin mediate hunger and when inhibited by the anorectic gut hormones PYY_3-36_ and GLP-1_7-36 amide_ mediate satiety.

The hypothalamus and certain brainstem nuclei are also known to be important in the homeostatic control of food intake. However, in a separate voxel-wise analysis, we did not observe significant responses in these regions with our visual stimulations (data not shown). Previous similar studies failed to observe changes in hypothalamic activation (Malik et al., 2008) or provided no comment on it (Farooqi et al., 2007), although other BOLD fMRI investigations have quantified hypothalamic signal (Batterham et al., 2007; Fletcher et al., 2010). Only one has reported on brainstem activation (Batterham et al., 2007). Approaches that control for effects of motion of brainstem during the cardiorespiratory cycle could enable the study of activation responses in future work (Pattinson et al., 2009). The small size of the hypothalamus (approximately 5 mm in diameter) limits its resolution using fMRI acquisition parameters applied in our study. Additionally, magnetic susceptibility signal loss due to the air-tissue interface of the adjacent sinuses limits assessment of any hypothalamic signal changes.

In summary, we have characterized the effects of single and combined administration of PYY_3-36_ and GLP-1_7-36 amide_ on brain BOLD fMRI activations in humans. We have shown that combined infusion of PYY_3-36_ and GLP-1_7-36 amide_ leads to an anorectic effect similar to that observed following a meal. In keeping with this, combined administration modulates brain activations implicated in appetite control to an extent similar to that observed physiologically after a meal. These findings provide direct evidence that the combined action of gut hormones including PYY_3-36_ and GLP-1_7-36 amide_ in the brain could explain postprandial satiety.

## Experimental Procedures

### Peptides

Synthetic human PYY_3-36_ and GLP-1_7-36 amide_ were purchased from Bachem (St. Helens, UK) and prepared for human administration as previously described (Neary et al., 2005).

### Subjects

Sixteen healthy right-handed subjects (11 male and 5 female, mean age 29.5 years, range 21–36 years, mean body mass index 22.1 kg/m^2^, range 18.3–25.1 kg/m^2^) were recruited through advertisement and assessed to be healthy during a screening visit with a full medical history, routine blood tests, and 12-lead electrocardiogram. Exclusion criteria were smoking, substance abuse, eating disorders, regular medication (except for oral contraceptives), pregnancy, and medical or psychiatric illness. The study was approved by the St. Mary's Research Ethics Committee (reference number 09/H0712/4) and performed in accordance with the Declaration of Helsinki. Full informed consent was obtained from all subjects prior to enrolment in the study. One male subject was excluded from the study after recruitment due to excessive head movement during the MRI scans, resulting in complete data from 15 subjects for analysis.

### Study Protocol

All subjects attended for six scan visits. Each visit was separated by at least 3 days. On their first visit, subjects always received an infusion of saline after an overnight fast. This protocol was identical to that used for the subsequent visits, and was intended to acclimatize the subject to the experimental procedures. Results from this acclimatization saline visit are not included in the analysis. During this acclimatization visit, a structural brain scan was obtained to exclude organic brain disease.

Over the following five study visits, following an overnight fast, subjects received each of the following interventions, in a single-blinded randomized fashion (Figure 1):

(1) A 90 min saline infusion (fasted saline, control visit).
(2) Standard breakfast, then a 90 min saline infusion (the fed saline visit).
(3) A 90 min PYY_3-36_ infusion at 0.3 pmol/kg/min.
(4) A 90 min GLP-1_7-36 amide_ infusion at 0.8 pmol/kg/min.
(5) A 90 min combined PYY_3-36_ and GLP-1_7-36 amide_ infusion at 0.3 pmol/kg/min and 0.8 pmol/kg/min, respectively.

The doses of PYY_3-36_ and GLP-1_7-36 amide_ were determined from previous human studies, which had shown that they would likely cause a significant reduction in energy intake but also would be well tolerated without side effects (Neary et al., 2005; Verdich et al., 2001).

Subjects fasted and drank only water from 22:00 hr the night before each study visit. They were asked to standardize their diet, abstain from alcohol, and avoid strenuous exercise for 24 hr prior to each visit. After arrival at 09:00 hr (t = −90 min), peripheral venous cannulae were inserted in both forearms (one for infusion and one for blood sampling). On their fed visit, subjects ate a standard 2500 KJ breakfast (see Energy Intake) in its entirety between 09:30 and 09:50 hr (t = −60 to −40 min). At 10:30 hr (t = 0 min), they were taken into the scanning room, and a 90 min infusion of saline or gut hormone commenced.

A MEDRAD MR Injector Spectris Solaris EP (Medrad, Indianola, PA) pump was used to deliver the infusions at a constant rate of 54ml/hr. Vials of peptide were dissolved in 2.5 ml vehicle. Blood samples were collected at t = −60, 0, 15, 30, 45, 60, 75, 90, and 120 min into lithium heparin-coated tubes containing 1500 kallikrein inhibitor units (0.15 ml) aprotinin (Trasylol, Bayer Schering Pharma, Berlin). Samples immediately underwent centrifugation, after which plasma was promptly separated and stored at −20°C until analysis.

At t = −60, 0, 90, and 120 min, subjects completed a series of 100 mm VAS that rated five food-related sensations (hunger, nausea, pleasantness to eat, how much one could eat, fullness) and four nonfood-related sensations (sleepiness, irritability, anxiety, and warmth).

The pulse and blood pressure of each subject was measured at t = −60, 0, 15, 30, 60, 75, 90, and 120 min. Blood glucose was checked on the samples taken at t = −60, 0, 30, 60, 90, and 120 min with an Optimum Exceed blood glucose monitor (Abbott Diabetes Care, Maidenhead, Berks).

Twenty minutes after the start of the infusion, a 60 min fMRI scan was performed. At the end of the scan, subjects stayed in the scanner room for a further 10 min until the infusion was stopped at t = 90 min. Subjects were then immediately served a meal that was provided in excess (see Energy Intake) and asked to eat until comfortably full. Water was freely available. Subjects could leave at t = 120 min.

### Energy Intake

The standard breakfast served to subjects during one of the fMRI study visits (2500 kJ) consisted of two medium slices of wholemeal bread, 20 g strawberry jam, 20 g margarine, two slices of Swiss cheese, 40 g bran flakes, 170 g milk, and 220 g orange juice. Subjects were asked to consume the entire breakfast within 20 min.

The ad libitum buffet meal was served in privacy for 20 min and consisted of either a mild chicken tikka curry with rice (628 kJ/100 g) or a vegetarian option of tomato and mozzarella pasta (507 kJ/100 g). Each subject had previously decided on their choice of meal during their screening visit and was subsequently served the same meal during each of their study visits. Food was weighed pre- and postconsumption. Energy intake was calculated from the weight of food consumed.

### Plasma Hormone Assays

PYY_3-36_ immunoreactivity was measured with a commercial radioimmunoassay (Millipore) (Adam et al., 2005).

Active GLP-1 (GLP-1_7-36 amide_ and GLP-1_7-37_) immunoreactivity was measured with a commercially available ELISA kit (Millipore) (King et al., 2011).

### Visual Analog Scores

Changes in VAS ratings on a 100 mm scale were assessed for each visit.

### Statistical Analysis

Grouped data are represented as the mean ± SEM. Comparisons of energy intake and ratings of meal palatability were by repeated-measures ANOVA with Tukey's multiple comparison posttest. VAS scores were adjusted for baseline and differences between t = 0 min and t = 90 min (the duration of the infusion) were compared by repeated-measures nonparametric Friedman's test with Dunn's multiple comparison posttest. Linear regression analysis was performed to assess correlation between nausea and energy intake over the whole study. Comparisons of plasma gut hormone levels during the infusion period (plasma samples from t = 15–90 min inclusive) were by repeated-measures ANOVA with Tukey's multiple comparison posttest. The threshold for statistical significance in each case was set at p < 0.05. Analyses were performed using Prism version 5.01 software (GraphPad Software, San Diego, CA).

### Functional MRI

T2^∗^-weighted, dual-echo, echo-planar images sensitive to BOLD contrast were acquired continuously on a 3T Siemens Tim Trio scanner with a 32-channel head coil (Siemens Healthcare, Erlangen, Germany).

### Functional MRI Picture Processing Task

During the fMRI picture processing task, images of foods and nonfood items were projected as previously described (Beaver et al., 2006).

### Functional MRI Analysis

Using FSL software (http://www.fmrib.ox.ac.uk/fsl/), we estimated the difference in regional mean BOLD signal intensity between periods of subject exposure to palatable food images in relation to balanced exposure to periods of nonfood images, at each of six prespecified ROIs bilaterally—amygdala, caudate, insula, nucleus accumbens, OFC, and putamen—using an in-house MRI atlas (Tziortzi et al., 2011). We computed the mean percent BOLD signal change across each ROI by scaling the relevant General Linear Model contrast parameter estimate with the peak-to-peak regressor amplitude and normalizing this “signal change” factor with the global image mean.

Further details about methodology of peptide administration, plasma hormone quantification, fMRI scanning, picture processing task, and analysis can be found in Supplemental Experimental Procedures.

## Supplemental Information

Document S1. Two Figures, Supplemental Experimental Procedures, and Supplemental References

Table S1. Local Maxima Where Δ BOLD, When Subjects Viewed Images of food Compared with When They Viewed Images of Nonfood, Was Significantly Greater Than Zero Imaging measures (cluster peaks meeting a criterion of p < 0.05, z > 1.6) for saline infusion (in the fed and fasted state) are shown. The response of each of the a priori ROIs to the picture processing task for saline infusion (in the fed and fasted state) is highlighted in bold in the second half of the table.
