# Supplementary material for: The Gut Hormones PYY3-36 and GLP-17-36 amide Reduce Food Intake and Modulate Brain Activity in Appetite Centers in Humans
Source: Cell Metab. 2011 Nov 2;14(5-2):700–6. doi: 10.1016/j.cmet.2011.09.010 (PMC3267038; doi:10.1016/j.cmet.2011.09.010)
Supplement: Document S1. Two Figures, Supplemental Experimental Procedures, and Supplemental References [file mmc1.pdf]

Supplemental Information
Cell Metabolism, *Volume 14*

The Gut Hormones $PYY_{3\text{-}36}$ and GLP-$1_{7\text{-}36\ amide}$ Reduce Food Intake and Modulate Brain Activity in Appetite Centers in Humans

Akila De Silva, Victoria Salem, Christopher J. Long, Aidan Makwana, Rexford D. Newbould, Eugenii A. Rabiner, Mohammad A. Ghatei, Stephen R. Bloom, Paul M. Matthews, John D. Beaver, and Waljit S. Dhillo

Figure S1

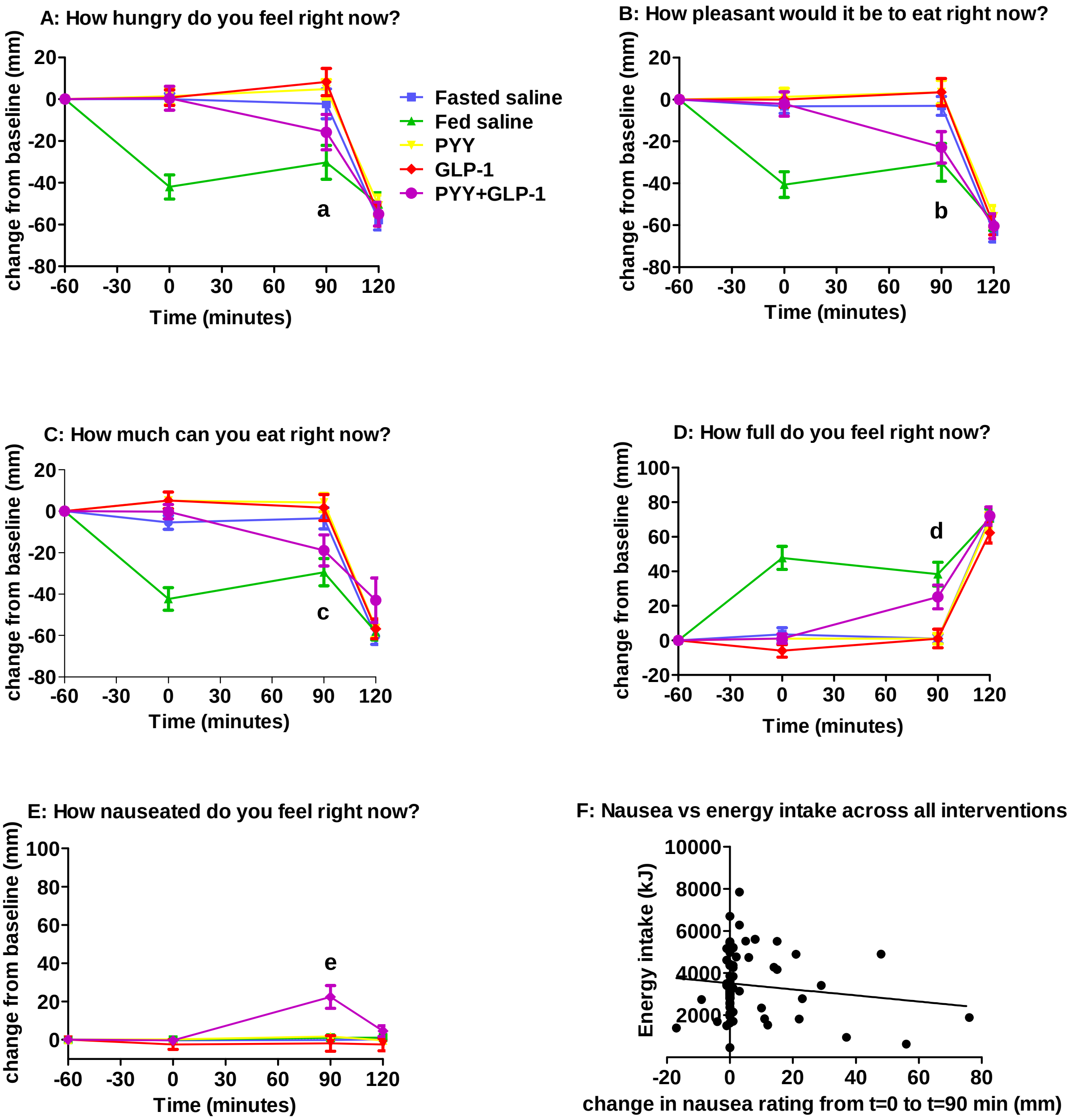

Figure S1, Related to Figure 2. Analysis of Food-Related Visual Analogue Score (VAS) Parameters from t = 0 to t = 90 min during Each Infusion

During each study, subjects completed 100 mm visual analogue scale questionnaires relating to hunger (A), pleasantness to eat (B), anticipated food consumption (C), fullness (D) and nausea (E). The change in VAS scores from baseline (t = 0 min) to the end of the infusion (t = 90 min) are shown. Data are shown as mean ± SEM. [a] denotes p= 0.03 for PYY+ GLP-1 vs. fed saline. [b] denotes p= 0.02 for PYY+ GLP-1 vs. fed saline. [c] denotes p= 0.007 for PYY+ GLP-1 vs. fed saline. [d] denotes p= 0.0002 for PYY+ GLP-1 vs. fed saline and p< 0.01 for PYY+ GLP-1 vs. fasted saline and PYY. [e] denotes p< 0.0001 for PYY+ GLP-1 vs. all other groups. p=ns for fasted saline vs. fed saline. Across all infusions, there was no correlation between nausea and energy intake (p= 0.23, r^2 = 0.02) (F). Abbreviations PYY = PYY_{3-36}, GLP-1 = $GLP\text{-}1_{7-36\ amide}$, PYY+ GLP-1 = combined infusion of PYY_{3-36} and $GLP\text{-}1_{7-36\ amide}$.

Figure S2

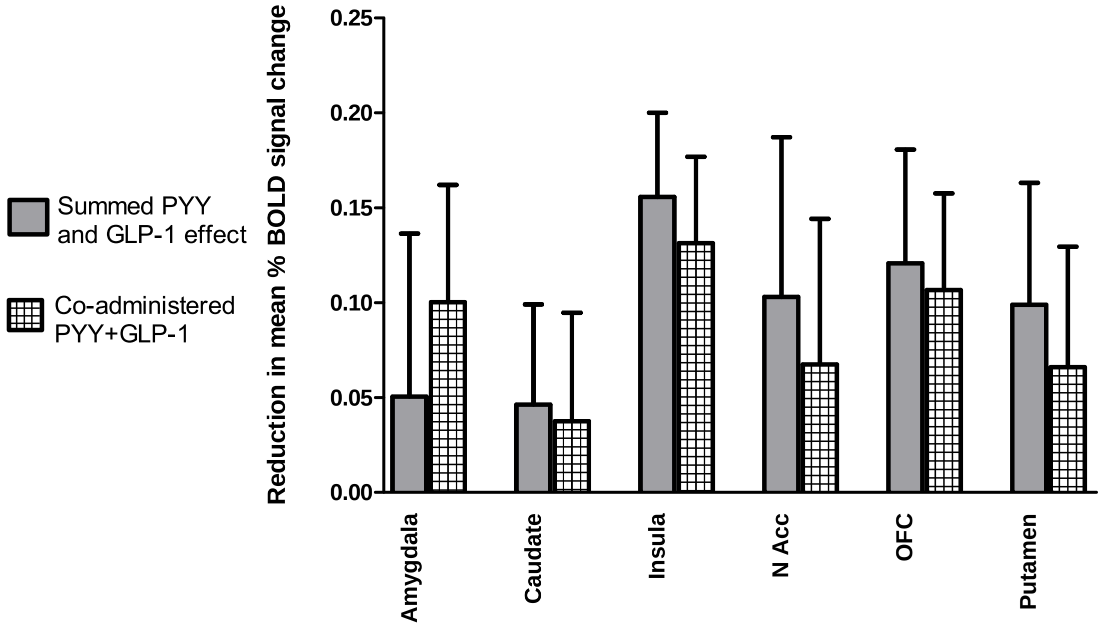

Figure S2, Related to Figure 4. Summed Reduction in Mean % BOLD Signal Change in ROIs with Individual Administrations of $PYY_{3\text{-}36}$ and GLP-$1_{7\text{-}36\ amide}$, Compared with the Reduction in Mean % BOLD Signal Change after Combined Administration of $PYY_{3\text{-}36}$ and GLP-$1_{7\text{-}36\ amide}$

The reduction in mean % BOLD signal change when subjects viewed images of food compared with when they viewed images of non-food (compared with fasted saline) is expressed as a sum of the individual effects of $PYY_{3\text{-}36}$ and GLP-$1_{7\text{-}36\ amide}$ (grey bars). This is compared with the reduction in mean % BOLD signal change when subjects viewed images of food compared with when they viewed images of non-food (compared with fasted saline) for the combination infusion of $PYY_{3\text{-}36}$ and GLP-$1_{7\text{-}36\ amide}$ (hatched bars). Data are shown for individual ROIs [amygdala, insula, caudate, nucleus accumbens (N Acc), OFC and putamen], combined for left and right hemispheres and are grouped for 15 subjects, shown as mean ± SEM. PYY= $PYY_{3\text{-}36}$; GLP-1= GLP-$1_{7\text{-}36\ amide}$ p= ns for all ROIs.

Please see Excel file for Table S1.

SUPPLEMENTAL EXPERIMENTAL PROCEDURES

Peptides

The identity and purity of each peptide was confirmed by matrix-assisted laser desorption ionisation-time of flight (MALDI-TOF) mass spectrometry, and by high performance liquid chromatography (HPLC) (Bachem). Peptides were dissolved in 0.9% sterile saline (Bayer, Haywards Heath, UK), aliquoted into vials and freeze dried. The vials were sterile on extended bacterial and fungal culture (Department of Microbiology, Hammersmith Hospital, London) and Limulus Amoebocyte Lysate test results (Associates of Cape Cod, Liverpool, UK) were within the safe range for human infusion. Toxicity studies were performed in mice and results were unremarkable (data not shown). During the human infusion studies, in order to reduce adsorption of peptide on to the walls of lines and syringes, all infusion sets were filled with Gelofusin (B. Braun Medical, Sheffield, UK) for 20 minutes, which was then discarded immediately prior to preparation of the peptide in the syringe. The vehicle used to dissolve and deliver peptide infusions was 10% Gelofusin in 0.9% saline.

Plasma Hormone Assays

The antibody used in the PYY radioimmunoassay fully cross reacts with only the 3-36 form of human PYY. There is no cross reactivity with the 1-36 form of the hormone. The Millipore $PYY_{3\text{-}36}$ assay utilizes ^{125}I labelled PYY and a $PYY_{3\text{-}36}$ antiserum to determine the plasma level of $PYY_{3\text{-}36}$ by the double antibody/PEG technique. The detection limit of the assay was 5 pmol/l, with an intra-assay coefficient of variation of 6.4-11.0 %. All samples were measured in one assay to avoid inter-assay variation.

In the GLP-1 ELISA, the monoclonal antibody immobilized in the wells of the microwell plate binds specifically to active forms of GLP-1 only ($GLP\text{-}1_{7\text{-}36\ amide}$ and $GLP\text{-}1_{7\text{-}37}$), with no cross reactivity with any other form of the hormone. Bound GLP-1 is conjugated to an anti-GLP-1

alkaline phosphatise, which produces the fluorescent product umbelliferone when methyl umbelliferyl phosphate is added. The amount of fluorescence generated is proportional to the amount of GLP-1 in the sample. The limit of detection was 2 pmol/l, with an intra-assay variation of 6-9 %. All samples were measured in one assay to avoid inter-assay variation.

fMRI scan

Each scan session consisted of 501 volumes of 36-slice acquisition, angled ~30º coronally to the anterior-posterior commissural plane to minimize signal dropout in orbitofrontal and medial temporal regions (TR = 2000 ms; dual TEs = 13 and 31 ms; flip angle = 80º, slice thickness= 3.0 mm, matrix size = 64 × 64, and field of view = 225 × 225 mm^2 for voxel size of 3.51 × 3.51 × 3.0 mm). High-resolution T1-weighted anatomical scans (ADNI MPRAGE (Jack et al., 2008)) were acquired with whole-brain coverage (208 slices) for each participant to facilitate fMRI image co-registration, and ROI definition (TR = 3000 ms, TE = 3.66 ms, flip angle = 9º, voxel size = 1 mm^3).

fMRI picture processing task

During the fMRI picture processing task, 75 images were shown, divided into three classes of 25 exemplars: 25 high-calorie foods, 25 low-calorie foods and 25 non-food items (Beaver et al., 2006). There were two sets of 75 images, alternated between each scanning session. Images from each category were presented in counterbalanced order across participants and sessions. The images appeared for 5 s, interspersed with periods of rest (fixation on a blue octagon), with each block lasting 25 seconds. Subjects were instructed to press a button in response to the images shown, and asked to alter the length of time they pressed the button depending on how pleasant they found each image.

fMRI analysis

fMRI data were preprocessed with FSL software (www.fmrib.ox.ac.uk/fsl/) in order to correct for motion, to register the echo planar functional images to the high-resolution anatomical (T1-weighted) scan of each individual, and to overlay images on a standardized atlas (MNI) in order to allow for comparisons across individuals. Data were high-pass filtered and spatially smoothed (5mm Full Width Half Maximum Gaussian kernel) to allow for gyral variability across subjects and to improve signal-to-noise ratio at the intra-subject level. Individual sessions with greater than 3.5mm absolute head motion or severe stimulus-correlated motion (as exhibited by marked rim artifacts in intra-subject statistic maps) were excluded from further analysis. This led to a total of 15 usable subjects with complete data from all infusion groups.

We estimated the difference in regional mean BOLD signal intensity between periods of subject exposure to palatable food images in relation to balanced exposure to periods of non-food images, at each of 6 pre-specified ROIs. All ROIs were defined as the conjunction between the full group main effect of task (food vs. non-food, using parametric testing at the level of spatially contiguous supra-thresholded clusters, while controlling the family-wise probability of type 1 error at $p<0.05$, corrected) and the relevant atlas region. We confirmed the accuracy of registration of ROIs on the BOLD functional data by visual inspection of their overlay in relation to the functional images i.e. in subject space. These estimates were then averaged over right and left homologous regions of amygdala, caudate, insula, nucleus accumbens, OFC, and putamen. For each infusion, we tested the null hypothesis that the within-subject difference between fasted saline, fed saline, PYY_{3-36} infusion, GLP-$1_{7-36\ amide}$ infusion or combined PYY_{3-36} + GLP-$1_{7-36\ amide}$ infusion in regional BOLD response to food images vs. non-food images was zero, i.e. [the gut hormone infusion or fed saline BOLD response to palatable food exposures] – [fasted saline BOLD response to palatable food exposures] = $\Delta BOLD = 0$. The statistical model for the primary study objectives was a

repeated measures analysis of difference of means, between control (fasted saline) and each infusion session's BOLD data. A paired t-test model was also used to test intra-regional difference effects of the three gut hormone infusion states and the fed saline state against fasted saline.

SUPPLEMENTAL REFERENCES

Beaver, J.D., Lawrence, A.D., van Ditzhuijzen, J., Davis, M.H., Woods, A., and Calder, A.J. (2006). Individual differences in reward drive predict neural responses to images of food. J Neurosci *26*, 5160-5166.

Jack, C.R., Jr., Bernstein, M.A., Fox, N.C., Thompson, P., Alexander, G., Harvey, D., Borowski, B., Britson, P.J., J, L.W., Ward, C., et al. (2008). The Alzheimer's Disease Neuroimaging Initiative (ADNI): MRI methods. J Magn Reson Imaging *27*, 685-691.
